# Lamarckian evolution of the giant Mimivirus in allopatric laboratory culture on amoebae

**DOI:** 10.3389/fcimb.2012.00091

**Published:** 2012-07-05

**Authors:** Philippe Colson, Didier Raoult

**Affiliations:** ^1^Unité de Recherche sur les Maladies Infectieuses et Tropicales Emergentes, Centre National de la Recherche Scientifique Unité Mixte de Recherche (UMR) 7278, Institut de Recherche pour le Développement (IRD) 3R198, INSERM U1095, IHU Méditerranée Infection, Facultés de Médecine et de Pharmacie, Aix-Marseille UniversityMarseille, France; ^2^Pôle des Maladies Infectieuses et Tropicales Clinique et Biologique, Fédération de Bactériologie-Hygiène-Virologie, Centre Hospitalo-Universitaire TimoneMarseille, France

**Keywords:** Mimivirus, Lamarckian evolution, Darwinian evolution, gene expression profile, transcription profile, genome reduction, allopatry

## Abstract

*Acanthamoeba polyphaga* Mimivirus has been subcultured 150 times on germ-free amoebae. This allopatric niche is very different from that found in the natural environment, where the virus is in competition with many other organisms. In this experiment, substantial gene variability and loss occurred concurrently with the emergence of phenotypically different viruses. We sought to quantify the respective roles of Lamarckian and Darwinian evolution during this experiment. We postulated that the Mimivirus genes that were down-regulated at the beginning of the allopatric laboratory culture and inactivated after 150 passages experienced Lamarckian evolution because phenotypic modifications preceded genotypic modifications, whereas we considered that genes that were highly transcribed in the new niche but were later inactivated obeyed Darwinian rules. We used the total transcript abundances and sequences described for the genes of Mimivirus at the beginning of its laboratory life and after 150 passages in allopatric culture on *Acanthamoeba* spp. We found a statistically significant positive correlation between the level of gene expression at the beginning of the culture and gene inactivation during the 150 passages. In particular, the mean transcript abundance at baseline was significantly lower for inactivated genes than for unchanged genes (165 ± 589 vs. 470 ± 1,625; *p* < 1e–3), and the mean transcript levels during the replication cycle of Mimivirus M1 were up to 8.5-fold lower for inactivated genes than for unchanged genes. In addition, proteins tended to be less frequently identified from purified virions in their early life in allopatric laboratory culture if they were encoded by variable genes than if they were encoded by conserved genes (9 vs. 15%; *p* = 0.062). Finally, Lamarckian evolution represented the evolutionary process encountered by 63% of the inactivated genes. Such observations may be explained by the lower level of DNA repair of useless genes.

## Introduction

**Figure 1:**
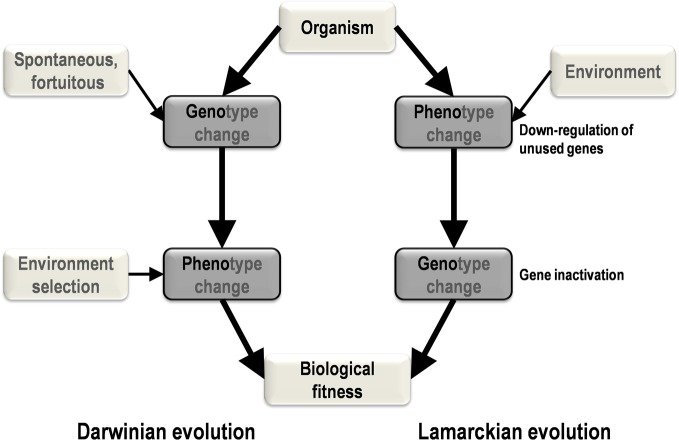
**A schematic diagram of the major steps and causes and effects in Darwinian and Lamarckian evolution**. In Darwinian evolution (left), genotypic change precedes phenotypic change, whereas these changes occur in the opposite order in Lamarckian evolution (right).

Two primary mechanisms of evolution, Lamarckian and Darwinian, have been generally recognized (Koonin, 2009; Koonin and Wolf, 2009). Among the elements that differentiate the theory of evolution of Lamarck (1809) from that of Darwin (1859) is the central Lamarckian concept that phenotypic changes result from adaptation to the environment and can be transmitted vertically. According to this view, phenotypic changes precede genotypic changes (Figure 1). In contrast, in the current vision of evolutionary biology and in accordance with the post-Darwinian modern synthesis, genetic modifications produce phenotypic changes and precede selection of the fittest in a given environment (Koonin, 2009). In this scenario, genotypic changes precede phenotypic changes.

**Figure 2:**
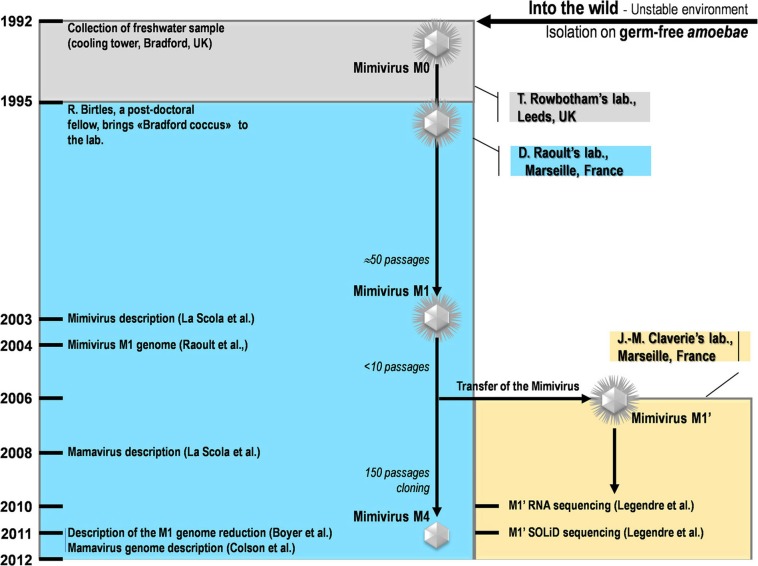
**A schematic diagram of the collection, isolation, and experiments conducted for Mimivirus and Mamavirus**.

**Figure 3:**
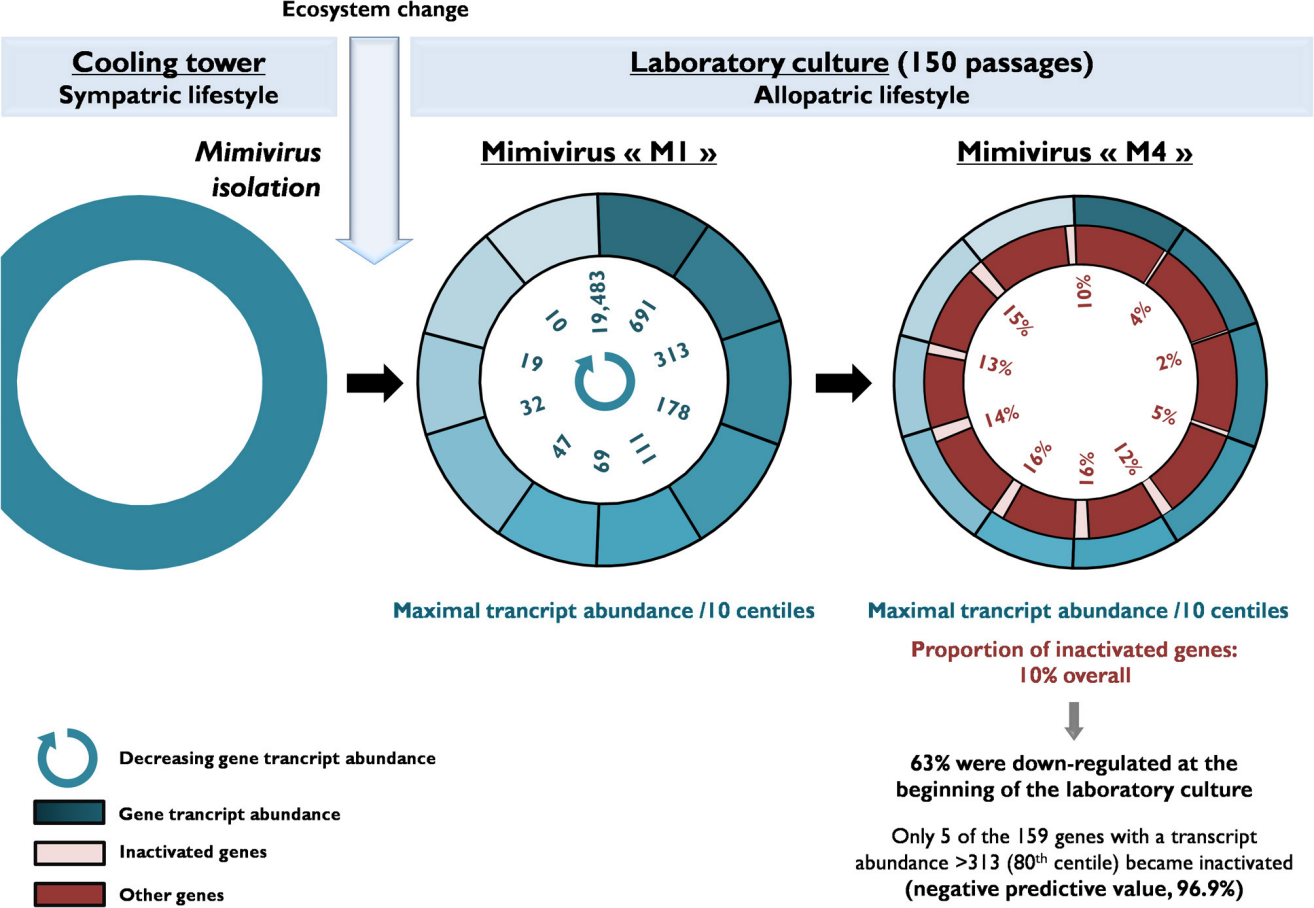
**A schematic diagram of the evolution of the gene content of Mimivirus during allopatric laboratory culture on amoebae**. The diagram shows the transcript abundance for Mimivirus M1 genes and the proportions of inactivated genes after 150 passages.

Our strain of *Acanthamoeba polyphaga* Mimivirus was recently subcultured 150 times on germ-free amoebae. This allopatric niche is very different from that found in the natural environment of Mimivirus, where the virus is in competition with many other organisms (Raoult and Boyer, 2010; Boyer et al., 2011; Figure 2). An interesting feature of this process of experimental evolution was the occurrence of Mimivirus gene variability and loss concurrently with the emergence of phenotypically different viruses (Boyer et al., 2011). The observed phenotypic changes included a lack of surface fibers and morphologically different viral factories compared with the first generation of wild-type Mimivirus at the beginning of the allopatric laboratory culture. In addition, another team had analyzed the transcriptome of this first generation of Mimivirus over its entire replication cycle (Legendre et al., 2010). These studies provided an opportunity to assess the respective roles of Lamarckian and Darwinian evolution in Mimivirus (Figures 1–3). Thus, we analyzed the nucleotide and amino acid variability of Mimivirus genes during the maintenance of the allopatric laboratory culture on amoebae by comparing their sequences in Mimivirus M4, recovered after 150 passages, with those in M1, recovered at the beginning of the *in vitro* culture (Figures 2 and 3). To quantify the apparent Lamarckian and Darwinian evolutionary processes in this experiment, we postulated that the inactivated genes of Mimivirus that had been down-regulated at an early phase in the new niche of the virus experienced Lamarckian evolution, as phenotypic modifications preceded genotypic modifications (Figure 1). Indeed, the regulation of gene transcription has been increasingly described in association with phenotypic changes that occur in life forms when they are introduced to a new biological and ecological niche (Revel et al., 2002; La et al., 2008; Smith and Kruglyak, 2008). An example of the dramatic alteration of gene expression was recently reported in a plant bacterium following host switching (Oshima et al., 2011). In contrast, inactivated Mimivirus genes that were normally transcribed during the early phase of life in the new niche were considered to obey Darwinian rules (Figure 1).

## Materials and methods

### Mimivirus M1 and M4 genes studied

The sets of genes for Mimivirus at the beginning of its laboratory life (Mimivirus M1) and after 150 passages in allopatric culture in *Acanthamoeba polyphaga* (Mimivirus M4) corresponded to those reported previously (Raoult et al., 2004; Boyer et al., 2011; Figure 2). BLASTn searches were performed with the Mimivirus M1 set of open reading frames (ORFs) against the Mimivirus M4 genome.

### Groups of genes defined based on their variability during allopatric laboratory culture

The genes present in Mimivirus M1 were classified into two major groups: group A is composed of genes that remained unchanged (i.e., showed 100% nucleotide identity) in the genome of Mimivirus M4, whereas group B includes Mimivirus genes showing variability at the nucleotide level during the allopatric laboratory culture (nucleotide identity <100%). The variable genes with a frameshift associated with a >30% size reduction of their product were considered to be inactivated. Group C is composed of Mimivirus M1 genes lost during the transition to the Mimivirus M4 genome, as indicated by the absence of significant BLASTn hits; these genes are located within two large fragments of the genome of Mimivirus M1 that have been deleted in Mimivirus M4. These two large deletions described by Boyer et al. have been considered to be “catastrophic” events that are neither Lamarckian nor Darwinian evolutionary processes, and they were, therefore, not included in our analysis (Boyer et al., 2011).

### Transcription and expression profiles of mimivirus M1 genes

The transcription profile of the first generation of Mimivirus corresponded to the transcript abundances determined by Legendre et al. at *T* = 0, 1.5, 3, 6, 9, and 12 h of the viral replication cycle (Legendre et al., 2010). These results were those for Mimivirus M1' and were considered to correspond to an early stage of the laboratory culture after Mimivirus moved from a sympatric niche to an allopatric niche (Figure 2). For each gene, the total number of normalized reads counts encompassing the different time points of the replication cycle was used (Legendre et al., 2010). The mean [±standard deviation (SD)] of gene total transcript abundance was 372 ± 1,342. The median value for all of the genes was 69. Gene expression was considered to be high at the beginning of the laboratory culture if the transcript abundance was equal to or greater than the median transcript abundance for all Mimivirus genes. Conversely, genes were considered to be weakly expressed at the beginning of the laboratory culture if the transcript abundance was lower than the median value for all genes. Centiles were calculated for the values of transcript abundance for all genes, and the proportions of unchanged and inactivated genes were calculated per groups of 10 centiles. The proportion of Lamarckian evolution was inferred from the proportion of Mimivirus genes weakly expressed at the beginning of the culture relative to the proportion inactivated after 150 passages in allopatric laboratory culture. Finally, the proteins cited as showing an association with Mimivirus M1 are those previously identified from purified virions by capillary LC-MS/MS, 2D gel electrophoresis, and MALDI-TOF mass spectrometry (Renesto et al., 2006).

### Comparison of the transcription profiles, expression, and variability of mimivirus M1 genes

We tested whether the transcription levels obtained for Mimivirus M1' genes at the baseline state of the laboratory culture predict the inactivation of these genes after 150 passages on germ-free amoebae (Figures 2 and 3). The proportions of highly and weakly transcribed genes, of the genes among the 25 most highly expressed or with transcript abundances above the 70th centile were compared between unchanged and variable or inactivated genes. In addition, the mean transcript abundance was compared among groups of genes defined on the basis of their variability during the allopatric laboratory culture. The correlations between the initial transcript abundance for each gene and the nucleotide variability or the number of nucleotide and amino acid positions that varied during the 150 passages were also studied. Moreover, both the transcript abundance and the number of differences between corresponding genes in Mimivirus M1 and M4 were plotted according to the location of the genes within the genome. For improved clarity, the mean values calculated for a sliding window of 10 genes and a step of 1 gene were presented with Microsoft Excel software. The number of A- or T-homopolymers with a stretch of ≥4 was determined for each Mimivirus gene. The occurrence of nucleotide differences between Mimivirus M4 and M1 flanking such a homopolymer was also assessed. In the statistical analysis of the data, proportions were compared with a corrected chi-square test or a Fisher exact test, and comparisons of means were performed with OpenEpi Epidemiologic Calculators v. 2.3.1 (www.OpenEpi.com). Linear regression was performed with MedCalc v. 11.6.1.0 (http://www.medcalc.org). *P* values <0.05 were considered to be statistically significant.

## Results

**Figure 4:**
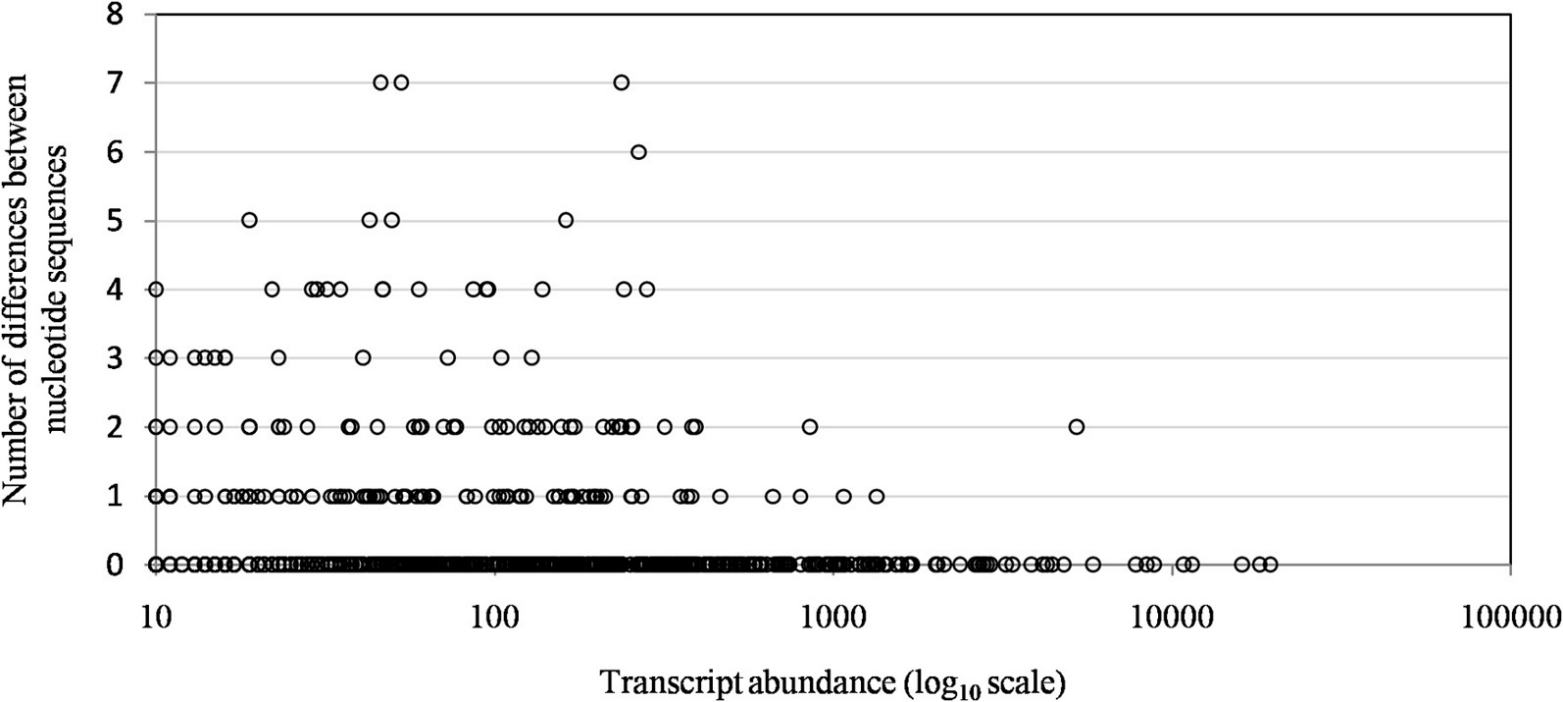
**The distribution of transcript abundance for Mimivirus M1 genes according to the number of differences between the nucleotide sequences of the Mimivirus M4 and M1 genes**. A difference corresponds to the presence of different nucleotides at the same position within a pairwise alignment provided by BLAST for the nucleotide sequences of corresponding genes of Mimivirus M1 and Mimivirus M4.

**Figure A1:**
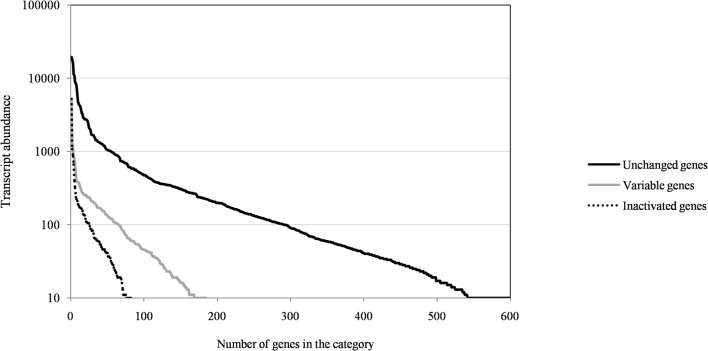
**The distribution of transcript abundance of Mimivirus M1 genes for unchanged, variable, and inactivated genes after 150 passages in allopatric laboratory culture on amoebae**.

Of the 960 genes present in Mimivirus M1, excluding those lost during the allopatric laboratory culture and representing large deletions, 606 (77%) were unchanged in the Mimivirus M4 genome (group A), and 185 (23%) were variable at the nucleotide level (group B) in the genome of Mimivirus M4. A total of 83 genes (10%) were considered to have been inactivated during the allopatric laboratory culture. The number of variable nucleotide positions between the same genes in Mimivirus M1 and M4 ranged between 0 and 7 (mean ± SD, 1.8 ± 1.3; Figure 4). The mean transcript abundance at baseline was significantly lower for inactivated genes than unchanged genes (165 ± 589 vs. 470 ± 1,625, respectively; *p* < 1e–3) and for variable genes than unchanged genes (141 ± 415 vs. 470 ± 1,625, respectively; *p* < 1e–3; Table 1; Figures A1 and 5). In addition, the mean transcript levels at different time points of the replication cycle of Mimivirus M1 were up to 8.5-fold lower for inactivated genes than unchanged genes (Figure 6). Moreover, the proportion of inactivated genes was significantly lower among the genes that were highly expressed at baseline than among those that were weakly expressed [7.7% (31/405) vs. 13.5% (52/386); *p* = 0.0077; relative risk (RR), 0.57 (95% confidence limits for RR (CI95), 0.37–0.87)] (Figure 3), and the proportion of variable genes was significantly lower among the genes that were highly expressed at baseline than among those that were weakly expressed [18.8% (76/405) vs. 28.2% (109/386); *p* = 0.0017; RR, 0.66 (CI95, 0.51–0.86)]. Otherwise, the proportion of genes with a transcript abundance greater than the 70th centile of the values for all genes (178) was significantly lower among the inactivated genes than among the unchanged genes [13.3 vs. 35.0%; *p* < 1e–3; RR, 0.38 (CI95, 0.22–0.66)]. This proportion was also significantly lower among the variable genes than among the unchanged genes [18.9 vs. 35.0%; *p* < 1e–3; RR, 0.29 (CI95, 0.19–0.46)]. The proportion of variable genes in the 25 genes most expressed at baseline tended to be significantly lower than the corresponding proportion of unchanged genes [0.5 vs. 3.5%; *p* = 0.034; RR, 6.4 (CI95, 0.87–47.34)]. The negative predictive value (NPV) of being inactivated was 92.3% for highly expressed genes. In addition, this NPV was 95.5 and 96.9% for genes with a transcript abundance greater than the 70th and 80th centile, respectively, of the values calculated for all genes. Furthermore, the proportion of genes encoding proteins identified from purified virions in their early life in allopatric laboratory culture tended to be lower in the variable than in the unchanged genes [9% (17/185) vs. 15% (88/606); *p* = 0.062; RR, 0.63 (CI95, 0.39–1.04)]; nine of the genes encoding proteins present in the virions were inactivated. Also, among the 23 class I–III core genes of the nucleocytoplasmic large DNA viruses (NCLDVs) that were not located within the large deletions observed in the Mimivirus M4 genome, 17 (74%) remained unchanged during the 150 passages on germ-free amoebae. Eleven (65%) of these 17 genes were highly expressed. In contrast, only one (4.3%) NCLDV core gene of classes I–III was inactivated; this latter gene was weakly expressed at baseline in the laboratory culture.

**Table 1:** **Comparative features for unchanged, variable, and inactivated Mimivirus genes after 150 passages in allopatric culture on amoebae**.

**Features of Mimivirus M1 genes**	**Group A: unchanged genes (*n* = 606)**	**Group B: variable genes**	
		**All (*n* = 185)**	**Inactivated genes (*n* = 83)**
Mean transcript abundance^a^	470 ± 1,625	141 ± 415^*^	165 ± 589^*^
Number of genes among the 25 most transcribed (%)	21 (3.5)	1 (0.5)^*^	1 (1.2)^*^
Number of genes with transcript abundance > the 70th centile for all genes (%)	212 (35)	35 (19)^*^	11 (13)^*^
Number of genes with transcript abundance ≤ the 10th percentile for all genes (%)	65 (10.7)	17 (9.2)	8 (9.6)
Proteins identified by proteomics (%)	88 (14.5)	17 (9.1)^†^	9 (11)

* *Proportion is significantly different from that of the genes of group A (p < 0.05; chi-square corrected test or Fisher's exact test)*.

† *Proportion tends to differ statistically from that of the genes of group A (0.05 < p < 0.1; chi-square corrected test)*.

a *From Legendre et al. (2010)*.

**Figure 5:**
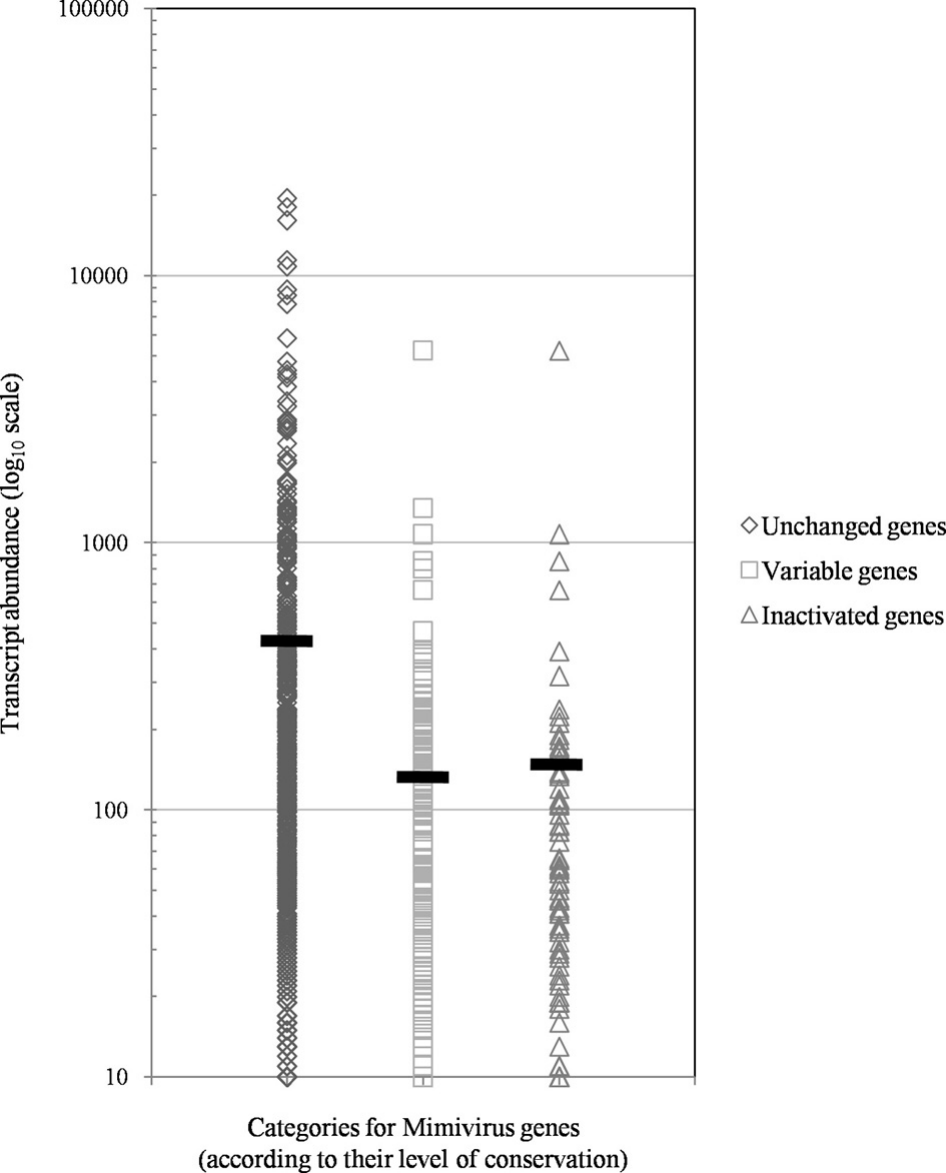
**The distribution of transcript abundance in Mimivirus M1 for different groups/subgroups of genes defined based on their variability and evolution during 150 passages in allopatric laboratory culture on amoebae**. The horizontal bars indicate the mean values for each group/subgroup.

**Figure 6:**
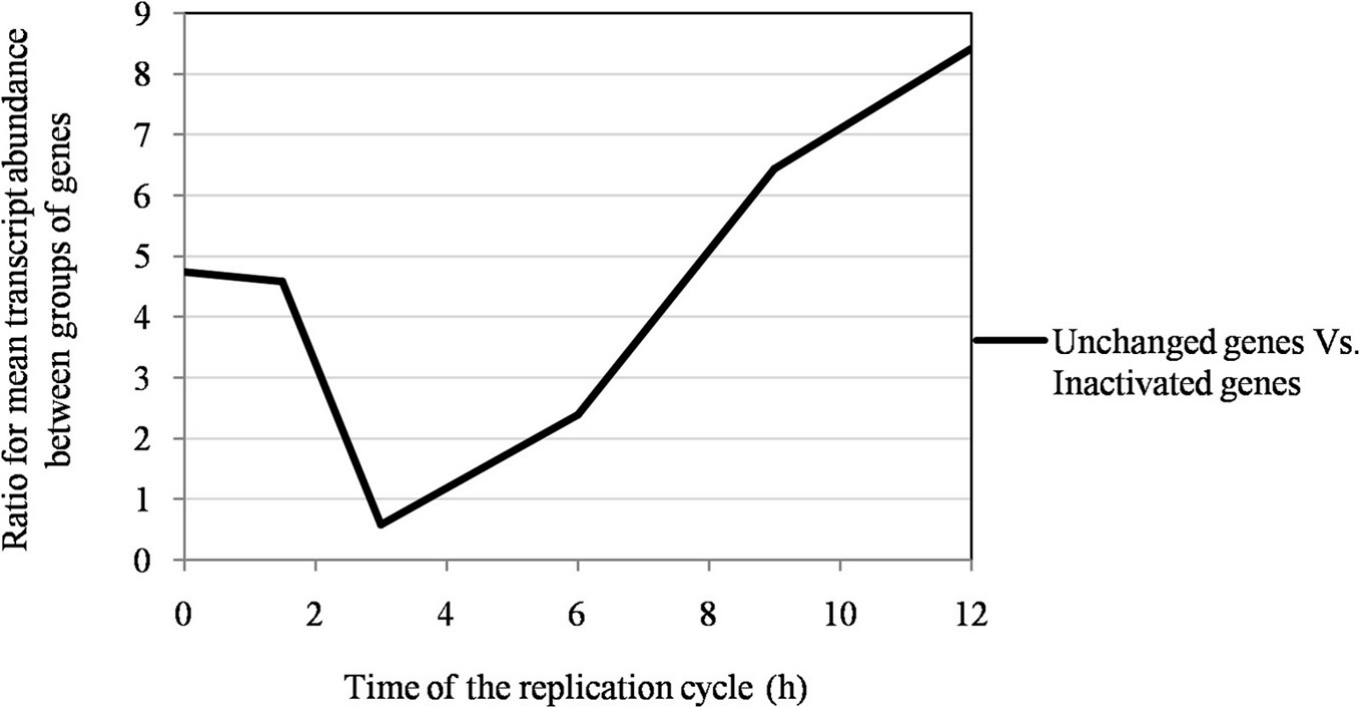
**The ratio of the mean transcript abundance in Mimivirus M1 between unchanged and inactivated genes during allopatric laboratory culture on amoebae**.

**Figure 7:**
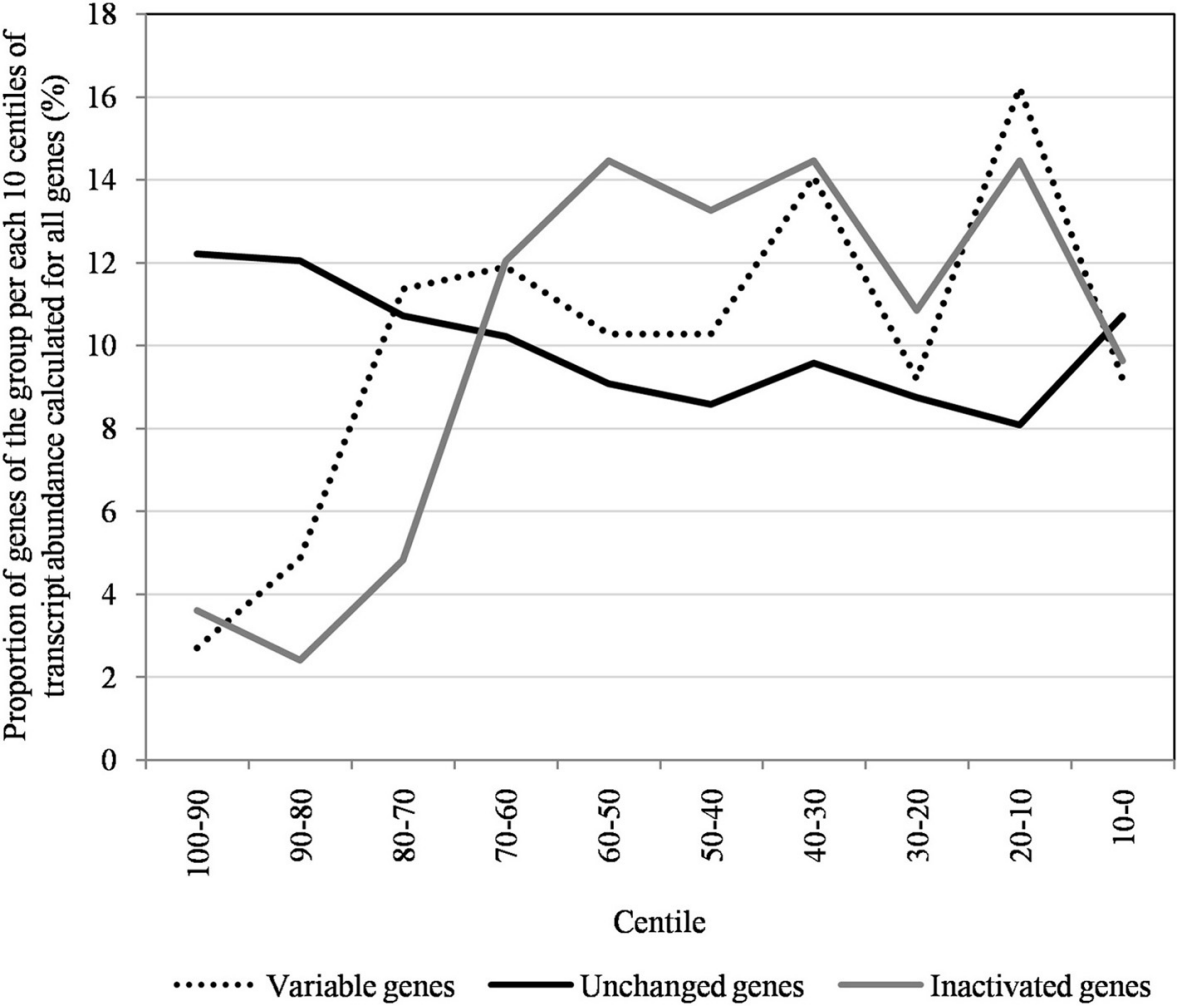
**The distribution of unchanged, variable, and inactivated genes after 150 passages in allopatric laboratory culture on amoebae per each 10 centiles of transcript abundance, calculated for all Mimivirus M1 genes**.

The proportion of inactivated genes was the lowest [2.4% (2/82)] for those with a transcript abundance between the 80th and 90th centiles (313–691). This proportion was < 5% above the 80th centile and >10% below the 80th centile (Figure 3). Moreover, the proportion of variable genes ranged from 6.3% (5/79) for those with a transcript abundance above the 90th centile to 38.0% (30/79) for those with a transcript abundance between the 10th and 20th centiles (Figure 3). Among the 79 Mimivirus M1 genes with a transcript abundance above the 90th centile, 5 (6%) were variable and 3 (4%) were inactivated. The three genes that were inactivated were two hypothetical proteins (R401, R750b) and an S/T protein kinase (R400). Conversely, among the 82 Mimivirus M1 genes with a transcript abundance below the 10th centile (<10), 17 (21%) were variable and 8 (10%) were inactivated. The distribution of genes according to their levels of transcript abundance showed very low proportions above the 80th centile for inactivated and variable genes, whereas the distribution of unchanged genes was homogeneous (Figure 7). Finally, the proportion of Lamarckian evolution, as defined by the proportion of genes weakly expressed at the beginning of the allopatric laboratory culture among those inactivated, was 63%.

**Figure A2:**
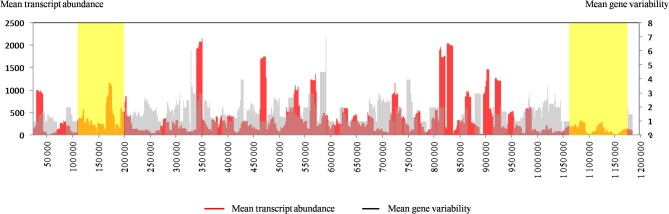
**The distribution along the Mimivirus M1 genome of the transcript abundance of Mimivirus M1 genes and the nucleotide variability for the same genes in Mimivirus M1 and M4**. For both parameters, the mean values for a sliding window of 10 genes and a step of 1 gene are represented. The variability was defined as the number of variable positions within genes. The yellow boxes toward the tips of the genome indicate large deletions encountered by the genome of Mimivirus during allopatric laboratory culture on amoebae, as previously reported (Boyer et al., 2011).

We sought to visually assess the relationship between gene transcription and nucleotide variability between the same genes in Mimivirus M1 and M4. For this purpose, we plotted the mean values for these two parameters along the genome according to a sliding window of 10 genes (step = 1) because representing the values for all genes did not allow sufficient legibility. As shown in Figure A2, this representation clearly indicates a strong inverse correlation between the initial transcript abundance and further gene variability. Thus, the regions of the genome composed of genes that were initially weakly expressed are those in which the genes showed the greatest variability. It might be hypothesized that mutations observed in the genome of Mimivirus M4 relative to Mimivirus M1 are the result of sequencing errors. Thus, the mean ± SD number of A- or T-homopolymers with a stretch of ≥4 per gene was 11.2 ± 9.1 (range, 0–79), and the mean ± SD number of nucleotide differences that flanked such homopolymers was 1.2 ± 1.0 (range, 0–6). Nevertheless, the mean number of homopolymers per 100 nucleotides was similar for the unchanged and variable genes, 1.0 ± 0.5 (range, 0–3) and 1.2 ± 0.3 (0–2), respectively.

This work is based on data obtained for Mimivirus at different stages of its early life under laboratory conditions (Figure 2). However, it is very unlikely that the genome sequences have been significantly affected by the initial number of subcultures on *Acanthamoeba* spp. Indeed, the genomes of Mimivirus M1, Mimivirus M1' and *Acanthamoeba castellanii* Mamavirus, another strain of Mimivirus, are highly similar although the viruses experienced different numbers of passages (from less than 10 to approximately 50) on *Acanthamoeba* spp. The comparison of the Mimivirus M1 genome with that recently recovered by ultra-deep sequencing of genomic DNA and total RNA on a SOLiD platform (Legendre et al., 2011), which we called Mimivirus M1', showed that the two sequences differ by only 196 substitutions, 29 deletions and 174 insertions. The comparison of the Mamavirus genome with the Mimivirus genome showed ≈99% nucleotide identity in their alignable regions, which represent nearly the entire length of these genomes. Moreover, the Mamavirus and Mimivirus pairs of genes with bidirectional best hits show a mean nucleotide identity of 98.8%, and the majority of the pairs have identity levels greater than 99%. Interestingly, among the 19 genes present in the Mimivirus genome and absent in Mamavirus that were considered in the present work (not located within the large deletions), 16 (84%) have been classified as weakly expressed, and 4 (25%) of these genes are among the inactivated genes. Moreover, among the 22 genes in which frameshifts were identified in a comparison of the Mimivirus and Mamavirus genomes, 15 (68%) were weakly expressed at baseline, and three (20%) were inactivated.

## Discussion

Our work shows that the majority (63%) of the genes inactivated during Mimivirus evolution in the allopatric laboratory culture was initially weakly expressed and that low gene expression at the beginning of the culture is significantly positively correlated with gene inactivation and variability during the 150 passages. A possible bias exists and is related to the initial stages of culture. Thus, Mimivirus M1 might have been selected from the original pool of viruses recovered from their natural environment due to a growth advantage when it was first moved to the laboratory culture environment, as is the case for every artificial system of culture. This issue has not been studied in our laboratory or by other investigators. However, the comparison of the genomes of Mamavirus (considered to be another strain of Mimivirus), Mimivirus M1 and Mimivirus M1' revealed very few differences despite differences in the number of passages on amoebae. In addition, the aim of the present work was to assess the capacity to predict genes that will be inactivated under stable laboratory conditions based on their initial transcription profile. In this context, we observed that a high level of expression for a gene strongly predicted its absence of inactivation. This finding suggests that the adaptation of Mimivirus to the modification of its environment after infection of germ-free amoebae *in vitro* is associated with a down regulation of certain genes that tended to be degraded and not repaired because they had become useless. A mechanism of this type, in which adaptation to a new ecosystem determines a new phenotype and this new phenotype promotes genotype changes transmitted to future generations, is the form of evolution described by Lamarck. One could consider that genotypic changes in the Mimivirus M1 genome were actually transmitted to new generations, as gene losses are usually considered irreversible (Krylov et al., 2003).

It was previously emphasized that the evolutionary rate of a gene sequence was negatively correlated with its level of expression or the abundance of its product (Pal et al., 2001; Koonin, 2011). This relationship was previously assessed by estimating the number of substitutions per nucleotide site between orthologous sequences in several lineages or organisms, but it has not been assessed for the same organism during experimental evolution, as is the case in the present work. In the same context, a positive correlation was recently described between the propensity for gene loss and a sequence's evolutionary rate and gene dispensability (Krylov et al., 2003). Moreover, it was found that highly expressed proteins evolve slowly (Drummond et al., 2005). In addition, it is known that DNA repair and damage processing particularly targets actively transcribed genes, as in the case of transcription-coupled repair (Hanawalt and Spivak, 2008). A similar process may explain the finding that down-regulated Mimivirus genes are more variable; they are less likely to be repaired than highly transcribed genes.

Lamarckian evolution may be involved in bacterial speciation events associated with a reduction of the genome size (Merhej et al., 2009), a finding opposed to the dominant model that considers that speciation and fitness gain are associated with an increase in gene repertoires. Thus, the major route of speciation (through adaptation to a given ecological niche) is typically through allopatry (Georgiades and Raoult, 2010) and is associated with genome size reduction through the loss of useless genes according to the mode described by Moran: “use it or lose it” (Moran, 2002). It is probable that such modifications of the gene repertoire are associated with the radical impossibility of returning to a previous ecosystem. Thus, in a noncompetitive environment during the 150 passages in allopatric culture, Mimivirus experienced a rapid and dramatic modification of its gene content that may substantially compromise its biological fitness in more complex environments. This observation is consistent with Ernst Mayr's vision of cause and effect in biology, in which the effects of changes differ in the short term and in the long-term (Mayr, 1961). In this case, we found that Mimivirus can be selected for rapid growth in an environment without competition. However, these changes prevent the virus from attaining fitness in competition with other intraamoebal organisms (Boyer et al., 2011). Therefore, the conservation of unused genes in allopatry is only important as a long-term strategy. Together with previous data, our results suggest that the transcriptome of Mimivirus may predict the evolution of its genome in a stable laboratory culture system and that Lamarckian evolution may contribute to the evolution of the Mimivirus genome in this environment. These findings offer an incentive to study the correlation between transcription profiles and the evolution of gene sequences and repertoires in particular organisms.

### Conflict of interest statement

The authors declare that the research was conducted in the absence of any commercial or financial relationships that could be construed as a potential conflict of interest.
